# Supplementary material for: Neural precursors of decisions that matter—an ERP study of deliberate and arbitrary choice
Source: eLife. 2019 Oct 23;8:e39787. doi: 10.7554/eLife.39787 (PMC6809608; doi:10.7554/eLife.39787)
Supplement: Supplementary file 1. [file elife-39787-supp1.docx]

Neural precursors of decisions that matter—an ERP study of deliberate and arbitrary choice

Maoz U, Yaffe G, Koch C, and Mudrik L

Supplementary Data

**Supplementary Table 1: NPO names and causes acronyms**

| **NPO** | **Cause** | **NPO website** |
| --- | --- | --- |
| Consensual NPOs | | |
| American Society on Aging | Pro Quality of Life for the Elderly | http://asaging.org/ |
| Conservation Fund | Pro Environment protection | http://www.conservationfund.org/ |
| Bill & Melinda Gates Foundation | Pro Education | http://www.gatesfoundation.org/ |
| Global Fund for Women | Pro Women's Rights | https://www.globalfundforwomen.org/ |
| The Hunger Project | Pro Hunger Relief | https://www.thp.org/ |
| Oxfam International | Pro Poverty & Disaster Relief | http://www.oxfam.org/ |
| World Wild Life Fund (WWF) | Pro Species Conservation | http://worldwildlife.org/ |
| Cancer Research Institute | Pro Cancer Research | http://www.cancerresearch.org/ |
| Habitat for Humanity | Pro Housing for All | http://www.habitat.org/ |
| Reading is Fundamental | Pro Advancement of Literacy | http://www.rif.org/ |
| International Institute for Conservation of Historic and Artistic Works | Pro Culture & Arts Preservation | https://www.iiconservation.org/ |
| Big Brothers and Big Sisters of America | Pro Youth Development | <http://www.bbbs.org/site/c.9iILI3NGKhK6F/>  b.5962335/k.BE16/Home.htm |
| United Nations Children's Fund (UNICEF) | Pro Child Protection | http://www.unicef.org/ |
| Doctors without Borders (Medecins sans frontieres) | Pro Disaster Medical Care | http://www.msf.org/ |
| Soldiers' Angels | Pro Veterans & Military | http://www.soldiersangels.org/heroes/index.php |
| Disability Rights International | Pro Disabilities Rights | http://www.disabilityrightsintl.org/ |
| National Crime Prevention Council (NCPC) | Pro Crime Prevention | <http://www.ncpc.org/> |
| Amnesty International | Pro Human Rights | https://www.amnesty.org/ |
| Peace Corps | Pro Peace & Development | http://www.peacecorps.gov/ |
| World Health Organization | Pro World Health | http://www.who.int/en/ |
| Controversial NPOs | | |
| Planned Parenthood | Pro Abortion & Family Planning | http://www.plannedparenthood.org/ |
| Pro-Life Alliance | Anti Abortion & Family Planning | http://www.prolifealliance.com/ |
| Human Rights Campaign | Pro LBGTQ Rights | http://www.hrc.org/ |
| National Organization for Marriage | Anti LBGTQ Rights | https://www.nationformarriage.org/ |
| Stem for Life Foundation | Pro Stem Cell Research | http://www.stemforlife.org/ |
| Christian Dental & Medical Association | Anti Stem Cell Research | http://www.cmda.org/ |
| Greenpeace | Pro Action Against Climate Change | http://www.greenpeace.org/international/en/ |
| Global Climate Scam | Anti Action Against Climate Change | http://www.globalclimatescam.com/ |
| National Association for Gun Rights | Pro Gun Rights | http://www.nationalgunrights.org/ |
| Coalition to Stop Gun Violence | Pro Gun Control | http://csgv.org/ |
| American Gas Association | Pro Fracking for Natural Gas | http://www.aga.org/Pages/default.aspx |
| Americans Against Fracking | Anti Fracking for Natural Gas | http://www.americansagainstfracking.org/ |
| StandWithUs (Israel) | Pro Israel | http://www.standwithus.com/ |
| Palestinian Centre for Human Rights | Pro Palestine | http://www.pchrgaza.org/portal/en/ |
| National Organization for the Reform of Marijuana Laws | Pro Marijuana Legalization | http://norml.org/ |
| Citizens Against Legalizing Marijuana | Anti Marijuana Legalization | http://www.calmca.org/ |
| Understanding Animal Research | Pro Scientific Experiments on Animals | http://www.understandinganimalresearch.org.uk/ |
| International Association Against Painful Experiments on Animals | Anti Scientific Experiments on Animals | http://www.iaapea.com/ |
| Federation for American Immigration Reform | Pro Immigration Reform | http://www.fairus.org/ |
| American Immigration Control | Anti Immigration Reform | http://www.immigrationcontrol.com/ |
| Human Cloning Foundation | Pro Human Cloning | http://www.humancloning.org/ |
| Americans to Ban Cloning | Anti Human Cloning | http://www.cloninginformation.org/ |
| Americans United for Separation of Church and State | Pro Separation of Church & State | https://www.au.org/ |
| Christian Coalition of America | Anti Separation of Church & State | http://www.cc.org/ |
| Death with Dignity National Center | Pro Euthanasia (Assisted Suicide) | http://www.deathwithdignity.org/ |
| Euthanasia Prevention Coalition | Anti Euthanasia (Assisted Suicide) | http://www.epcc.ca/ |
| The Alliance for Better Foods | Pro Genetically Modified Foods | http://www.betterfoods.org/ |
| Non-GMO Project | Anti Genetically Modified Foods | http://www.nongmoproject.org/ |
| Answers in Genesis | Pro Creationism Teaching | https://answersingenesis.org |
| National Center for Science Education | Pro Evolution Teaching | http://ncse.com/ |
